# Non-invasive Neurophysiology in Learning and Training: Mechanisms and a SWOT Analysis

**DOI:** 10.3389/fnins.2020.00589

**Published:** 2020-06-05

**Authors:** Angelica M. Tinga, Tycho T. de Back, Max M. Louwerse

**Affiliations:** Department of Cognitive Science and Artificial Intelligence, Tilburg University, Tilburg, Netherlands

**Keywords:** learning, training, neurophysiology, brain activity, heart rate, eye tracking, skin conductance, respiration

## Abstract

Although many scholars deem non-invasive measures of neurophysiology to have promise in assessing learning, these measures are currently not widely applied, neither in educational settings nor in training. How can non-invasive neurophysiology provide insight into learning and how should research on this topic move forward to ensure valid applications? The current article addresses these questions by discussing the mechanisms underlying neurophysiological changes during learning followed by a SWOT (strengths, weaknesses, opportunities, and threats) analysis of non-invasive neurophysiology in learning and training. This type of analysis can provide a structured examination of factors relevant to the current state and future of a field. The findings of the SWOT analysis indicate that the field of neurophysiology in learning and training is developing rapidly. By leveraging the opportunities of neurophysiology in learning and training (while bearing in mind weaknesses, threats, and strengths) the field can move forward in promising directions. Suggestions for opportunities for future work are provided to ensure valid and effective application of non-invasive neurophysiology in a wide range of learning and training settings.

## Introduction

While behavioral methods, such as pre- and post-test assessments, are most commonly used to assess learning, non-invasive neurophysiological methods provide promising complementary options (Leff et al., 2011; Lai et al., 2013; Krigolson et al., 2015; Tinga et al., 2019a). Non-invasive neurophysiology includes measures that provide insight into the nervous system through relatively non-invasive sensors placed on the body or in the environment. Examples of these measures are heart rate, electrodermal activity (EDA) and electroencephalography (EEG).

Although many scholars deem these measures to be promising in assessing learning, they are currently not widely applied in educational settings and in training. This article aims at providing insights that help the field move toward valid and effective application of neurophysiology in a wide range of learning and training settings. In the current article learning is considered as processing of information from experiences in order to update system properties (Barron et al., 2015). Through experience the ability to execute tasks or operations or processing of information improves and becomes faster, less effortful and more automatic (Schneider and Chein, 2003; Borghini et al., 2016). A large body of cognitive and neurocognitive studies demonstrates that learning manifests itself in different ways, such as the acquisition of new information, the development of perception, reasoning, psychomotor skills, and problem solving skills (Lai et al., 2013). When referring to learning in the current article we specifically refer to psychomotor and cognitive learning, but generalize to other types of learning. Affective learning, such as fear conditioning and fear extinction, however, is not included as this type of learning falls outside of the scope of the current article, as studies on this topic primarily reflect the affective response to stimuli. To gain insight into how we can move in the direction of valid and effective application of neurophysiology in a wide range of learning and training settings, it is first of all important to know the underlying mechanisms of how non-invasive measures of neurophysiology are able to inform on learning. To this aim we will first discuss the most frequently applied non-invasive measures and into what aspects of the nervous system these measures provide insight. Subsequently, we will discuss how activity in the nervous system, in turn, can provide insight into learning processes.

The nervous system consists of the autonomic and central nervous system. The autonomic nervous system has two branches that operate at the same time, but in an antagonistic way: the sympathetic nervous system and the parasympathetic nervous system, in which the sympathetic branch activates a neurophysiological response, while the parasympathetic branch inhibits one (Berntson et al., 1991), with parasympathetic nerves exerting their effects faster than sympathetic ones (Nunan et al., 2010). The nervous system regulates a wide variety of functions related to processes such as homeostasis, digestion and attention (Berntson and Cacioppo, 2000; Mauss and Robinson, 2009).

Non-invasive methods providing insight into the autonomic nervous system include measures of peripheral physiology such as heart rate (variability), respiration, EDA and electromyography (EMG). Heart rate and heart rate variability (i.e., the variation in the intervals between consecutive heartbeats) can be measured either via electrocardiography (ECG) or photoplethysmography (PPG). While ECG is more conventional, PPG has the benefit of being relatively less invasive. With ECG multiple sensors are placed on the body to measure the heart’s electrical activity, while with PPG a single optical sensor is placed on the finger or earlobe in order to measure peripheral changes in blood flow which are affected by activity of the heart. Although ECG is considered to be more precise than PPG, especially in measuring heart rate variability, the two measures are highly correlated under ideal conditions (Lu et al., 2009). Respiration can be measured through a respiration belt around the upper body measuring the body’s expansion and contraction. EDA refers to variations in the electrical properties of the skin in response to sweat secretion. A non-invasive measure of the change in conductance of the skin can be acquired by applying a low constant voltage across two electrodes placed on the skin (Fowles et al., 1981). EMG provides insight into muscle activity as this method measures electrical activity that is generated by muscle fibers. While the heart and the lungs are connected to the sympathetic as well as to the parasympathetic nerves, the skin and its sweat glands and skeletal muscles are solely innervated by the sympathetic nerves (Roatta and Passatore, 2008; Setz et al., 2010).

The central nervous system refers to the part of the nervous system consisting of the brain and the spinal cord. Brain activity can be measured non-invasively through measures such as EEG and functional near-infrared spectroscopy (fNIRS). fNIRS is a method in which cortical hemodynamic activity is measured using near-infrared light and is based on the assumption that neural activation and the vascular response are coupled (León-Carrión and León-Domínguez, 2012). EEG records cortical electrical activity non-invasively through electrodes on the scalp (Antonenko et al., 2010). EEG is applied more often than fNIRS, as fNIRS is still a relatively new measurement technique (Kopton and Kenning, 2014). Compared to EEG, fNIRS has a better spatial resolution but a reduced temporal resolution (Crosson et al., 2010; Zama and Shimada, 2015). Other well-established techniques of measuring brain activity include functional magnetic resonance imaging (fMRI) and magnetoencephalography (MEG). However, these measures tend to be more invasive (Tinga et al., 2019a) and therefore fall outside the scope of the current review.

Eye-related measures are another category of neurophysiological measures. The eye is affected both by the central and autonomic nervous system; the retina is an integral part of the central nervous system, while the muscles that control eye-movements, eyelid elevation and pupil dilation are innervated by both branches of the autonomic nervous system (McDougal and Gamlin, 2015; Eckstein et al., 2017; Larsen and Waters, 2018). Eye-related measures can be collected non-invasively through an eye-tracker either placed on a table in front of or mounted on the head of the trainee. Alternatively, sensors can be placed on the skin close to the eyes to measure the electrical activity that is paired to the eye’s position and movements, a technique called electrooculography (EOG). Eye-related measures include (1) pupil dilation; (2) eye gaze; (3) blink rate; and (4) blink duration (McDougal and Gamlin, 2015; Eckstein et al., 2017). Why would the activity in the nervous system measured through neurophysiology be related to learning processes? Measures of neurophysiology respond in a predictable manner to cognitive demand/mental workload or mental effort (Gevins and Smith, 2003; Antonenko et al., 2010; Setz et al., 2010; Ayaz et al., 2012; Brouwer et al., 2012; Borghini et al., 2014; Hogervorst et al., 2014; Reiner and Gelfeld, 2014; Gable et al., 2015; Charles and Nixon, 2019). As mental effort increases, sympathetic activation increases and parasympathetic inhibition decreases (Berntson et al., 1991), paralleled by changes in the central nervous system such as in oscillations in the EEG signal with alpha oscillations generally increasing and theta oscillations generally decreasing with decreasing demands (Klimesch, 1999; Klimesch et al., 2005; Antonenko et al., 2010; Brouwer et al., 2012) and in activation patterns in the fNIRS signal with activity in the (pre)frontal cortex generally increasing (Ayaz et al., 2012; Hiyamizu et al., 2014).

According to several influential cognitive theoretical frameworks learning can be thought of as a change in how a to-be-learned task is processed and therefore in how cognitively demanding a task is. The dual processing theory constitutes one of these frameworks. From the dual processing perspective, learning can be thought of as a transition from controlled to automatic processing (Schneider and Chein, 2003). Controlled processing is slow, effortful and attentionally demanding and is comparable to a high mental effort. Automatic processing, in contrast, is fast and occurs in the absence of control and attention and is comparable to low mental effort. Evidence for the transition from slow controlled processes to faster automatic processes comes from behavioral findings demonstrating that the beginning of the learning process is characterized by slow and often inaccurate responses (Schneider and Shiffrin, 1977) and that when learning advances the time needed for task execution decreases (Ritter and Schooler, 2001).

Similarly, from the perspective of cognitive load theory learning can be seen as the process of schema acquisition and schema automation (Schnotz and Kurschner, 2007), whereby items that were first processed separately become integrated within one schema and regarded as a single item. In this way, fewer items need to be held in working memory, reducing mental effort. Processing of the acquired schema can in turn become automatic during further learning. This schema automation reduces working memory demands or demands on cognition even more.

Mental effort additionally increases with increased task difficulty (Backs and Seljos, 1994; Hockey, 1997; Brouwer et al., 2014) and task difficulty is thought to modulate learning-related changes in neurophysiology (Wang et al., 2010). In general, execution of a task becomes less difficult during learning, eliciting changes in mental effort and therefore neurophysiology (Fairclough et al., 2005). Moreover, according to motivational intensity theory mental effort is proportional to the level of task demand provided that success is possible and successful performance is worthwhile (Brehm and Self, 1989). This has been demonstrated on several neurophysiological measurements as well (Richter et al., 2008, 2016; Fairclough and Ewing, 2017), implying that success needs to be possible in order for learning to occur and for mental effort and neurophysiology to change in a predictable way during learning.

The topic of learning consists of various aspects of development in cognition. It is important to take into account that different aspects of learning progress differently and mostly do not affect cognitive development in a linear fashion (Schneider and Chein, 2003; Luft and Buitrago, 2005; Murre, 2014; Stoianov et al., 2016). Even though cognitive development does not necessarily progress in a linear fashion, it is clear that important cognitive changes take place during learning, transitioning from more controlled and demanding processing to less demanding automation, which can be detected by neurophysiological measures. Experimental evidence comes from studies demonstrating changes in neurophysiology suggesting a decrease in mental effort over time during learning (for an overview of 113 experiments from 2006 to 2016 and a first model based on the findings from these experiments see Tinga et al., 2019a). Generally, in such experimental studies, a learning task is presented in which improvements over time (e.g., trials or blocks) on behavioral outcomes occur and the effect over time on simultaneously recorded neurophysiological measures is assessed and related to the behavioral learning process. For example, in a recent study including two experiments (Tinga et al., 2020) participants were presented with a visuomotor sequence learning task while behavioral measures and measures of EEG, skin conductance, heart rate (variability) and respiration were collected. Results indicated that, in both of the two experiments, skin conductance level and EEG oscillations (in the alpha and gamma band) changed during general task learning indicating less mental effort over time and were related to behavioral performance. These findings suggest neurophysiology is able to provide robust insights in learning. Moreover, several studies have compared learning and non-learning task conditions and report a clear difference on neurophysiology (Krigolson et al., 2009; Alain et al., 2010; Takeuchi et al., 2011; Moisello et al., 2013; Tan et al., 2014), supporting that there is potential in these measurements as they are able to dissociate learning from non-learning.

Different measures of neurophysiology all provide insight into activity in a part of the nervous system, yet there are important differences in specific challenges associated with each measurement technique. For example, measurements of heart rate variability are sensitive to whether the trainee is sitting, standing or lying down (Acharya et al., 2005). As another example, measures of pupil size are affected by the luminance of the surroundings (Höfle et al., 2008). Even though important differences between the different neurophysiological measures exist, we will consider all measures together in this review. These different measures of neurophysiology are rarely considered together, while it has been pointed out that it is important to discover what specific aspects of the learning process are reflected in specific neurophysiological changes (Tinga et al., 2019a). Moreover, different measures of neurophysiology can potentially be combined to provide better insight into learning than a single measure (cf. Wilson and Russell, 2003, 2007). However, where needed, important differences between different measures of neurophysiology will be discussed.

Even though neurophysiological measures have potential to provide insight into learning, the question remains how research on this topic should move forward to ensure valid applications. The nature of this current overview does not allow to go into the details of methodological issues inherent to the studies reviewed, but will instead assume the importance of these peer-reviewed articles and focus on how to best move the field forward.

## A Swot Analysis for the Field of Non-Invasive Neurophysiology in Learning and Training

To gain a better understanding of how the field of non-invasive neurophysiology can best move forward, it is important to comprehensively assess the current state of the field. To this aim, we will identify strengths, weaknesses, opportunities, and threats (SWOT) for the field of non-invasive neurophysiology in learning and training. Although the SWOT framework is most commonly employed in business for analyzing factors that influence a company’s position in the marketplace with a focus on the future, it can also be useful for other domains such as in a scientific field (Rizzo and Kim, 2005). Before we discuss each of the strengths, weaknesses, opportunities and threats, we present a summary of the analysis as a guideline (Table 1).

**TABLE 1:** Summary of the SWOT analysis for the field of non-invasive neurophysiology in learning and training, with each of the items discussed in the text.

Strengths	Weaknesses
• Formative assessment providing insight into the process of learning • Supports predicting the outcome of learning • Objective assessment • Supporting learning through instruction or feedback • Tailoring learning task to the individual in real-time • Multi-channel data collected at a high sample rate • Deeper insights into learning through rich data • Flexible and precise application and measurement	• Neurophysiology reflects a wide variety of functions supported by the nervous system • Neurophysiological changes during learning are sensitive to individual differences and task-related aspects • Signal noise • Limited freedom of movement of trainee (wired/uncomfortable equipment) • Technical challenges (synchronization and analysis of large amounts of data from multiple data streams) • Personnel training, device maintenance, preparations, and procedures needed for high quality data • Hardware and software expenses

**Opportunities**	**Threats**

• The potential of neurophysiology in learning has been pointed out by multiple scholars • The research field is highly interdisciplinary, bringing knowledge together from experts from different fields • General changes in neurophysiology do not require precisely time-locking signals to repeated exposure of specific events, allowing for application in a wider range of contexts • Solutions for dealing (automatically) with noise • Real-time data analysis and visualization • Development of adequate wireless sensors (e.g., wearables) that take less set-up time and are more comfortable to wear • Reduction of costs • Neurophysiological sensors embedded in learning systems/widely used devices/promising new technologies	• Mostly applied in controlled lab environments using carefully structured tasks with simple stimuli • Focus has been mainly on specific learning topics; unclear how generalizable current findings are across different learning tasks • Focus has been mainly on specific neurophysiological measures; unclear how generalizable current findings are across a wide range of measures • Doubts about the validity and applicability of neurophysiological measures in learning have been raised • Ethical challenges such as data privacy • Trainee can feel uncomfortable being measured through sensors • Non-experts tend to trust conclusions from neurophysiological data even if they are unwarranted

### Strengths in Applying Neurophysiological Measures in Learning and Training

#### Formative and Objective Assessment Supporting Predicting the Outcome of Learning

Learning can be assessed in a summative or formative way. Summative assessment focuses primarily on assessing the outcome of learning while formative assessment aims to gain insight into learning processes (Stobart, 2008). While summative assessment informs on whether learning is sufficient, formative assessment additionally allows for supporting learning based on the examination of the learning process itself. Learning is commonly evaluated using summative measures of behavioral performance by for example using a *post hoc* written examination (Weinstein, 2001) focused on the outcome of learning while neglecting the learning process itself. Having insight into the learning process could even support predicting the outcome of this process even before someone has finished learning (Cowley et al., 2013; Vecchio et al., 2019).

Additionally, assessment needs to be objective, valid, and reliable (Brenner et al., 2015). *Post hoc* behavioral measures might lack objectivity and validity as they might be affected by the process of measurement itself with participants changing their behavior on purpose or without being conscious about it when they are able to detect that specific behaviors are being measured (Page et al., 1966). Non-invasive neurophysiological measures can be an interesting substitution or addition to behavioral outcomes of learning by being a formative assessment tool providing insight into the learning process online in an objective way.

#### Supporting Learning Through Instruction or Feedback and Tailoring Task to the Individual

Providing feedback during learning can greatly benefit learning (Hattie and Timperley, 2007; Sigrist et al., 2013). As neurophysiology is a formative measure, it can be employed for real-time support of learning. When measures indicate that the trainee is having difficulty with learning, the trainer can provide additional instruction and feedback. Potentially, automated feedback as a response to incoming neurophysiological data and additional task data could even be implemented in the learning task itself. The continuous stream of objective data could also be used for personalization of the learning environment to the needs of the individual in order to ensure that learning is neither too easy nor too difficult. For example, the learning task could be made more challenging when the trainee is learning sufficiently, but when learning is insufficient more challenging material could be presented at a later point in time or the task could be made simpler. Walter et al. (2017) studied this principle using EEG data during arithmetic learning in students. They adapted the difficulty of the material to the learner’s workload and concluded that such an adaptive learning environment is feasible. Additionally, systems are being developed in which eye tracking data is employed in order to track learning and support learning through feedback and adaptation in e-learning environments (Barrios et al., 2004; Song et al., 2015).

#### Multi-Channel Data Collected at a High Sample Rate Providing Rich Data

Non-invasive neurophysiology allows for measuring through multiple channels within one modality as well as across a range of modalities. Channels can be combined in order to reach conclusions of enhanced reliability as it has been demonstrated by Wilson and Russell (2003, 2007) that mental effort could be classified using a combination of neurophysiological measures (however, see Hogervorst et al., 2014; Tinga et al., 2020 who did not find that combining different neurophysiological measures improved assessment of mental effort and learning specifically). Besides the potential of enhancing reliability, if one channel fails to record sufficient data there are other channels to fall back on. Additionally, data can be collected at a high sample rate during the learning experience. For example, the sample rate typically ranges from 250 to 2000 Hz for EEG (Reis et al., 2014), from 25 to 2000 Hz for eye tracking (Reis et al., 2014), and from 500 to 1000 Hz for heart rate (Kwon et al., 2018). Although a higher sample rate does not always provide extra information (Reis et al., 2014; Kwon et al., 2018), a relatively higher sample rate allows for a precise description of processes over time. Data provided by frequently sampled multi-channel neurophysiological methods is therefore much richer than a single *post hoc* behavioral measure such as an exam score.

#### Flexible and Precise Application and Measurement

Another benefit of neurophysiological methods is that there is flexibility in where each sensor will be placed with multiple ways in which one modality can be measured while still recording with high precision. When measuring brain activity through EEG or fNIRS for example, one can decide to place sensors only at specific locations on the scalp or on locations distributed over the scalp. EEG sensors have an outstanding temporal resolution providing insights in the temporal dynamics of neuronal activity below 100 ms. Additionally, EEG sensors allow for source localization with adequate precision, especially from 64 electrodes, with a higher number of electrodes not being necessary (Baillet et al., 2001; Michel et al., 2004; Michel and Murray, 2012). For applications other than source localization, it is not required to include a large number of electrodes for measuring EEG, with studies reporting successful mental effort assessment through a single EEG sensor (Mak et al., 2013; Hogervorst et al., 2014). Regarding measurements such as heart rate and EDA, different configurations of electrode placement are possible (Francis, 2016), providing flexibility across a range of tasks. For example, heart rate, while traditionally measured via sensors on the chest, can alternatively be measured on the ankle/foot (Díaz and Pallas-Areny, 2010; Jarchi and Casson, 2016). As EDA works by measuring the change in conductance of the skin (Fowles et al., 1981), several different placement locations are possible. Cameras collecting eye tracking measures can be integrated into glasses worn by the trainee or can be positioned at a distance from the trainee (Meißner et al., 2019), as long as the cameras can reliably detect the eyes. Alternatively, sensors can be placed on the skin close to the eyes (EOG). Thus, there is a lot of flexibility in the type of sensors and the location of placements of the sensors, with measurements being able to be highly precise when they are suited to the goal of the measurements.

### Weaknesses in Applying Neurophysiological Measures in Learning and Training

#### Neurophysiology Is Reflective of a Wide Variety of Functions Supported by the Nervous System

Besides the important strengths associated with applying non-invasive measures of neurophysiology in learning and training, there are also important weaknesses. One weakness is inherent to the general-purpose nature of the nervous system. Its activity is not reflective of one process, but rather of a wide variety of functions related to homeostasis, digestion, attention, and so forth (Berntson and Cacioppo, 2000; Mauss and Robinson, 2009), which is an important point to keep in mind when interpreting measures reflective of nervous system activity. Although learning affects neurophysiology and these effects have been detected across several studies (Krigolson et al., 2009; Alain et al., 2010; Takeuchi et al., 2011; Moisello et al., 2013; Tan et al., 2014), neurophysiology is additionally sensitive to many learning-irrelevant processes and events. For example, pupil size is determined to a great extent by luminance of the surroundings making it important to control for luminance when interpreting pupil size data (Höfle et al., 2008). Effects of ambient light have also been reported for other neurophysiological measures, such as heart rate, with effects depending on the time of day (Ruger et al., 2006). Additionally, effects of emotion and stress (Mauss and Robinson, 2009) and mental fatigue (Gergelyfi et al., 2015) on measures of neurophysiology have been reported frequently. As learning is a process involving prolonged cognitive activity, mental fatigue can be induced in the trainee. Learning and mental fatigue are thought to both progress over time and therefore disentangling the two is important in order to determine which changes in neurophysiology are truly reflective of learning. Gergelyfi et al. (2015) experimentally demonstrated (as the first to do so according to the authors) that pupil size, skin conductance, heart rate, and EEG were unaffected by mental fatigue while blink rate and heart rate variability were affected by mental fatigue. These findings suggest that different neurophysiological measures are affected by mental fatigue in different ways, although more research is needed in order to be able to deal with effects of mental fatigue sufficiently.

#### Neurophysiological Changes During Learning Are Sensitive to Individual Differences and Task-Related Aspects

Changes in neurophysiological outcome measures during learning are also affected by individual differences and aspects of the learning task (Tinga et al., 2019a). For example, several studies have demonstrated changes in brain activity as assessed through EEG during learning to be different between young and older adults (Alain and Snyder, 2008; Eppinger et al., 2008; Pietschmann et al., 2008, 2011; Eppinger and Kray, 2011). Effects of age on learning-related changes in neurophysiology have not yet been studied for other outcome measures than measures of brain activity. It therefore is unclear whether age also affects measures of peripheral physiology. Neurophysiological changes during learning are also reported to be affected by the sensory system in which learning takes place, the number of learning days, and whether feedback on performance is provided (Tinga et al., 2019a). However, when controlling for individual differences and for task-related aspects, effect sizes for measuring learning through neurophysiological outcome measures become as large as effect sizes for measuring learning through behavioral outcome measures. This means that even though the sensitivity of neurophysiology might make assessment somewhat more complicated, neurophysiology can provide just as powerful insights in learning as behavior. Potentially, the sensitivity of neurophysiological outcome measures to individual differences and task-related aspects could even provide for valuable insights into learning processes in addition to insights obtained through behavioral measurements.

#### Signal Noise and Limitation of Freedom of Movement of the Trainee

Non-invasive neurophysiological measures can be noisy, especially outside of the controlled environment of the lab. Most techniques provide the most valid outcomes under ideal conditions. Commonly encountered noise sources are electrode motion and muscle contraction artifacts, usually caused by movements of the trainee (Vazquez et al., 2012; Poungponsri and Yu, 2013). The sensitivity to motion and muscle activity can be problematic for techniques using electrodes placed on the body, especially when measuring signals of lower amplitudes, such as EEG. When collecting eye-related measures using an eye tracker, muscle and motion artifacts are non-existent as no electrodes are placed on the body of the trainee, while other sources of noise can arise such as movements of the eye tracking camera with respect to the eye and changes in corneal reflection (Li et al., 2008). To deal with artifacts, in research on neurophysiology in learning the trainee is generally presented with a seated task and instructed to remain seated as still as possible. This brings us to another weakness, namely that of the limitation of freedom of movement of the trainee, which can, besides by being induced by the design of the task, be induced by neurophysiological measuring equipment worn by the trainee. Most such equipment is wired, and, depending on the type of measurement collected, can be uncomfortable to wear (Liao et al., 2012), affecting the trainee’s mobility.

#### Technical Challenges and Personnel Training

Technical challenges also arise when applying neurophysiology in learning and training, as collection, synchronization, and analysis of large amounts of data from multi-channel data streams are required (Dahlstrom-Hakki et al., 2019). Technological advancements are making these requirements increasingly easier, yet overcoming these technical challenges as much as possible is time-consuming as things such as time desynchronization between multiple devices and delays between the device and scripts occur frequently (Dahlstrom-Hakki et al., 2019). Sufficient attention and time should be given to personnel training and device maintenance and extra time should also be allocated for experimental preparations and procedures during testing to ensure that data of high quality is collected (Kivikangas et al., 2011).

#### Hardware and Software Expenses

Besides the costs associated with the time-investment in personnel training, maintenance of devices and experimental preparations and procedures, hardware, and software for non-invasive neurophysiological measurements can also be expensive. For example, costs of state-of-the-art eye tracking equipment range between US$ 20,000 and US$ 30,000 (Ferhat and Vilariño, 2016) and EEG equipment can cost more than US$ 75,000 (Krigolson et al., 2017). Costs of sensors measuring peripheral physiology such as heart rate, respiration and EDA can also be high (Lucena et al., 2015), with a complete research system including hardware and software easily reaching US$ 10,000 per modality.

### Opportunities for Applying Neurophysiological Measures in Learning and Training

#### Potential Is Recognized and the Field Is Highly Interdisciplinary

Applying non-invasive measures of neurophysiology opens up many opportunities. Multiple scholars have pointed out the potential of neurophysiology in learning and training. For example, Lai et al. (2013) reviewed 113 studies evaluating the effect of learning in general on eye-related outcome measures and indicated that they are promising in assessing learning within various themes with various topics. Regarding research on brain activity in learning, a review of about 90 studies on EEG changes during motor learning (Krigolson et al., 2015) concluded specific event-related brain potentials (ERPs) are able to provide important insights into learning. Additionally, Leff et al. (2011) assessed over 80 studies on brain activity measured via fNIRS during motor tasks and pointed out that this modality is promising for studying motor learning. Tinga et al. (2019a) reviewed 113 experiments examining a wide range of non-invasive neurophysiological measures during learning (including measures of brain activity, eye-related outcome measures, measures of heart rate, skin conductance and respiration) and demonstrated that effect sizes were large for neurophysiological changes during learning, although they were smaller than for behavioral changes. Yet, the difference in effect sizes between neurophysiology and behavior disappeared when controlling for task-related aspects and individual differences. Again, the authors concluded that neurophysiology is promising for the assessment of learning. Although most work on neurophysiology in learning is conducted in the controlled environment of the lab, research has also demonstrated neurophysiological measures can be successfully applied for examining learning in real-life scenarios such as in a classroom (Ko et al., 2017; Poulsen et al., 2017; Bevilacqua et al., 2019; Dahlstrom-Hakki et al., 2019).

The research field of neurophysiology in learning and training is also highly interdisciplinary, which allows for combining knowledge from experts in fields such as sensor technology, signal processing, artificial intelligence, neurophysiology, and engineering. Of course, it is a challenge to oversee and bring together the research in all these areas of expertise (Brouwer et al., 2015), but when one brings together people from these different areas we believe that this will greatly benefit formulating solutions.

#### Employing General Changes in Neurophysiology

The feasibility of applying measures of neurophysiology outside the lab increases, when examining changes in neurophysiology reflective of general changes in mental effort, instead of examining specific responses to specific stimuli as is the case for example with ERPs from EEG. Specific responses can only be computed when a task itself presents many similar stimuli that can elicit an ERP that can be reliably time-locked to those stimuli, or when a task-irrelevant probe can be repeatedly presented during learning (Gevins and Smith, 2003). Therefore, measures of more general changes in neurophysiology over time that do not need to be precisely time-locked after repeated exposure of a specific event, such as changes in frequency bands in EEG, pupil size or heart rate, are presently better suited for application in real-world learning and training contexts, opening up many possibilities.

#### Solutions for Dealing With Noise and Real-Time Data Analysis and Visualization

As discussed above, neurophysiological techniques generally provide the most valid outcomes under ideal conditions. Yet, solutions for dealing (automatically) with noise are available and are being enhanced (Vorobyov and Cichocki, 2002; Vazquez et al., 2012; Poungponsri and Yu, 2013), increasing validity and applicability. The rapid developments in machine learning contribute to the processing (e.g., enhancing signal to noise ratio) and analysis (e.g., exploiting multiple modalities) of neurophysiological measures (Müller et al., 2008; Lemm et al., 2011).

Moreover, there are several ways in which neurophysiological data can be analyzed, with some applied studies having proposed a simple form of analysis. For example, in analyzing eye tracking data one can look at the percentage of visual attention allocation across regions of interest in the visual field, which is easier to perform even with eye-trackers with a lower sample rate and facilitates interpretation (Dahlstrom-Hakki et al., 2019). Yet, simplifying analyses can come at the cost of having less deep insight into collected data than more advanced analysis techniques. Advanced analyses are however becoming more accessible through the increased availability of powerful software tools. Examples of relatively frequently used open source software tools for aiding in analyzing brain activity signals are FieldTrip (Oostenveld et al., 2011), Brainstorm (Tadel et al., 2011), and MNE (Gramfort et al., 2013).

Neurophysiological signals can also be analyzed and visualized in real-time. For example, it is possible to analyze EEG data online taking into account individual variations (Guger et al., 2000) and to visualize the data using open-source software (Mullen et al., 2013). Another example is real-time analysis and monitoring of neurophysiology via a cloud system which has shown to be accurate and effective for ECG (Xia et al., 2013). These advancements aid the ease and speed at which data can be assessed and support providing the trainee with effective feedback and tailoring the learning experience to the individual.

#### Development of Wireless Sensors and Reduction of Costs

Advancements to enhance the trainee’s comfort and ease of movement are also being made with wireless and more compact sensors already being made available. For example, wearable wristbands such as the Empatica E4 and the Everion (Biovotion) are able to measure multiple peripheral neurophysiological outcomes and systems such as B-ALERT (Advanced Brain Monitoring) and g.Nautilus (g.tec) provide wireless EEG measures. These newer devices require considerably less set-up time and are less sensitive to motion artifacts. Sensors are even becoming fully portable with the possibility for data collection to occur via a smartphone or tablet, even for measures of brain activity (Stopczynski et al., 2014; Poulsen et al., 2017). All these devices promise to provide medical grade data and several studies have provided evidence of their successful application (Amaral et al., 2017; Corino et al., 2017; Barrios et al., 2018; Beeler et al., 2018; Tinga et al., 2019b).

In general, wearables and more applied systems are substantially cheaper, easily being 5–20 times less costly to purchase. Together with the reduction of set-up time and advanced solutions for dealing with noise and enhancing data analysis, the total costs involved for being able to apply neurophysiology in learning and training are greatly reduced.

However, the performance of these systems is in many instances still not on par with more traditional lab-based systems due to limitations such as a lower number of channels, less precise application and lower sample rate (Hairston et al., 2014; Melnik et al., 2017). Yet, we expect technological advancements in the upcoming years to substantially reduce the difference in data quality between lab-based systems and wearables, making these wearables increasingly interesting to apply.

#### Embedding Sensors in Learning Systems and Widely Used Devices

Another interesting direction for future developments is to embed equipment measuring neurophysiology in learning systems. Such systems could then potentially be applied to diagnose learning states and provide instantaneous help or adapt the material based on the recorded neurophysiological data (Lai et al., 2013). One category of systems that has high potential in education and training is virtual reality (de Back et al., submitted) and existing virtual reality headsets and to-be developed headsets with integrated eye tracking and brain activity sensors can contribute to this potential (Jantz et al., 2017). As an example, Sood and Singh (2018) proposed a framework on combining virtual reality and EEG in an educational game for robust and resilient learning with an enhanced quality of the learning experience.

Additionally, integrating sensors in smartphones, laptops, and tablets might also be an interesting endeavor, with for example advancements being made in using cameras of such devices for collecting eye-related measures (Lin et al., 2013; Lim et al., 2015; Krafka et al., 2016; Meißner et al., 2019) and measures of heart rate and heart rate variability (Heathers, 2013; Lucena et al., 2015).

### Threats for Applying Neurophysiological Measures in Learning and Training

#### Measures Are Mostly Applied in Lab Settings Using Controlled Tasks

Although applying neurophysiology in learning and training brings along interesting possibilities, there are important threats involved that also need to be considered. To start with, studies examining neurophysiology in learning are mostly fundamental, being conducted in the controlled environment of the lab using carefully selected tasks with simple stimuli (Dahlstrom-Hakki et al., 2019; Tinga et al., 2019a). This fundamental work is obviously important to understand in what way and under what circumstances neurophysiology provides insight into learning. Yet, if we want to be able to assess and enhance learning in the real-world, outside of the lab, we would benefit from combining this fundamental knowledge with more applied knowledge.

#### Focus on Specific Learning Topics and Specific Neurophysiological Measures

The majority of studies on non-invasive neurophysiology in learning have focused on brain activity and eye-related outcome measures, while relatively few studies examined other outcome measures such as skin conductance, heart rate, EMG, and respiration (Leff et al., 2011; Lai et al., 2013; Krigolson et al., 2015; Tinga et al., 2019a), making it unclear how well current findings generalize to these other outcome measures. Additionally, most work has focused on perceptual learning and the acquisition of new information or conceptual knowledge (Lai et al., 2013; Tinga et al., 2019a). Some learning topics should be explored in more detail in order to ensure generalizability across a wide range of tasks (Krigolson et al., 2015). Therefore, more research is needed examining a wider range of neurophysiological measures on a range of tasks.

#### Validity and Applicability of Neurophysiology in Learning and Training

Although many scholars have deemed neurophysiology as having high potential in assessing learning and training, some important concerns about validity and applicability have been raised by another group of scholars. Brouwer et al. (2014) pointed out changes in neurophysiology over time during learning might not be specifically related to the learning process itself, but rather reflect unspecific changes. Brouwer et al. (2014) demonstrated that multiple neurophysiological measures (i.e., EEG, ECG, EDA, respiration, and eye-related measures) were sensitive to difficulty level but that there was no interaction between difficulty level and time on task during learning. Similarly, Cowley (2015) argued neurophysiology is able to measure simple cognitive states, but is limited in measuring higher-order cognition. Ansari et al. (2011) even argue that it is shortsighted and unrealistic to expect that results from research in cognitive neuroscience will have a direct and immediate impact in education. Dahlstrom-Hakki et al. (2019) also indicated that we are still far from being able to use neurophysiology to directly measure learning or knowledge, with these tools at best allowing us to make inferences about a complex process such as learning by studying it during carefully structured tasks. Concerns like these raise the question how valid and applicable these measures are in assessing learning, especially outside the environment of the lab. Indeed, most of the literature measuring neurophysiology during task performance focuses on measuring and predicting simple cognitive states such as arousal or attention, without examining learning (Charles and Nixon, 2019).

#### Ethical Challenges and Feeling Uncomfortable Being Measured Through Sensors

The collection of a large quantity of psychological data originating from the body of a trainee obviously brings along ethical challenges. Such a large dataset on bodily signals might contain sensitive information in addition to effects of learning. For example, neurological, respiratory, and cardiac disorders are known to influence neurophysiological measures (Willems et al., 1991; Daly and Wolpaw, 2008). Even someone’s physical and psychological health affects neurophysiology (Lovallo, 2011; Iorfino et al., 2016). One needs to ensure that this data will not be used for unethical purposes.

Additionally, trainees might feel uncomfortable being measured through sensors, with the sensors potentially inducing fear. For instance, Neven (2015) discussed the effects of a home monitoring system for the elderly consisting of cameras and sensors. Neven (2015) described that one woman developed “sensor phobia”, an intense fear of sensors. Albeit an extreme example, the fear of sensors alongside the ethical issues pose a threat to applying neurophysiological measures, including those for learning and training.

#### Non-experts Perception of Warrantability of Neurophysiology

A final threat is one related to the perception of the layman audience. Non-experts tend to regard neurophysiology to convey an objective truth, even if conclusions based on neurophysiology are unfounded. For example, Weisberg et al. (2008) found that a group of non-experts judged explanations of phenomena containing brain-based information as more satisfying than explanations without this information, even when that information was logically irrelevant. Especially bad explanations were rated more satisfying when irrelevant neurophysiological information was added. When it comes to neurophysiology in learning and training specifically, educational concepts based on neurophysiology can be popular even when claims are not backed by scientific evidence (Howard-Jones, 2009). These results indicate that one needs to be careful that non-experts are not misled by explanations using neurophysiological information. Researchers and practitioners should therefore ensure they are clear about what can presently be concluded from their results and what might become possible in the future (Brouwer et al., 2015).

## Conclusion

Considering the various aspects of the SWOT analysis, it is apparent that the field of neurophysiology in learning and training is developing rapidly with many opportunities existing and emerging. In these developments, weaknesses should be taken into account, such as the fact that neurophysiology responds to a wide variety of functions supported by the nervous system, and technical challenges related to synchronization and analysis of large amount of data. We should also be aware of the threats that are involved, which includes the fact that research has mainly been conducted in controlled lab environments focusing on specific learning topics and specific neurophysiological measures, as well as ethical challenges and trainees possibly feeling uncomfortable being measured through sensors. Yet, neurophysiology in learning and training has powerful and valuable strengths, such as allowing for formative and objective assessment, tailoring the task to the individual, and gaining deeper insights through multi-channel data. Additionally, there are several opportunities for the field of neurophysiology in learning and training.

To start with, as the associated costs to record neurophysiological measures continue to go down and equipment becomes less intrusive for the trainee and can be set-up more quickly and easily, applying non-invasive neurophysiology in learning and training will become increasingly feasible. We expect technological advancements in the upcoming years to substantially reduce the difference in data quality between lab-based systems and wearables. The advancements will allow for focusing on examining effects in more detail outside of the lab to make sure that current results established in the lab replicate in more applied and less controlled environments. Moreover, it will become more feasible to assess a wider range of measures to add to the literature which is currently mostly focused on measures of brain activity and eye-related outcomes.

The important challenge to distinguish learning-specific effects from other effects will become easier to deal with when research has identified under which conditions effects will hold for which neurophysiological measures, in combination with advancements in data quality and dealing with noise. Especially when taking advantage of the highly interdisciplinary nature of the research field, experts can work together in advancing toward solutions to dealing with challenges like these.

As in any other field, ethical issues such as proper data handling should be considered carefully. These issues will especially be apparent as application of non-invasive neurophysiology in learning increasingly takes place outside the lab. One possible direction could be to take advantage of real-time data analysis and visualization of more general changes in neurophysiology, which does not necessarily involve saving a full dataset with specific, and possibly sensitive, responses. This online assessment of neurophysiological effects could already provide trainers, and even trainees, with important information on the learning process and can be used to tailor the learning task in real-time.

Also, one should ensure findings in the field are communicated clearly and acknowledge limitations in order to warrant that findings are not misinterpreted. Here one could also take advantage of the highly interdisciplinary nature of the research field, which allows for addressing a wide range of aspects to guarantee a comprehensive view of new findings.

Developments in including neurophysiological sensors in learning systems and new technologies such as virtual reality systems will open up many interesting possibilities for application. For example, promising immersive learning experiences can be more effectively adapted to the individual when making use of online (automatic) formative assessment through neurophysiology.

Opportunities of neurophysiology in learning and training can be seized most effectively by having insight into the weaknesses and threats of their application as well as their unique strengths. In this way, the field of non-invasive neurophysiology in learning and training can move forward in promising directions getting closer toward valid application of neurophysiology in a wide range of learning and training settings.

## Author Contributions

All authors: conceptualization, and writing-review and editing. AT: writing-original draft preparation. ML: funding acquisition.

## Conflict of Interest

The authors declare that the research was conducted in the absence of any commercial or financial relationships that could be construed as a potential conflict of interest.
